# Reduction of malaria vector mosquitoes in a large-scale intervention trial in rural Burkina Faso using *Bti* based larval source management

**DOI:** 10.1186/s12936-019-2951-3

**Published:** 2019-09-14

**Authors:** Peter Dambach, Till Baernighausen, Issouf Traoré, Saidou Ouedraogo, Ali Sié, Rainer Sauerborn, Norbert Becker, Valérie R. Louis

**Affiliations:** 1 Institute of Public Health, University Hospital Heidelberg, 69120 Heidelberg, Germany; 2 Centre de Recherche en Santé de Nouna, Nouna, Burkina Faso; 3 German Mosquito Control Association (KABS), 67346 Speyer, Germany

**Keywords:** *Bacillus thuringiensis israelensis*, Vector control, *Anopheles*, Larval source management, Burkina Faso

## Abstract

**Background:**

Malaria remains one of the most important causes of morbidity and death in sub-Saharan Africa. Along with early diagnosis and treatment of malaria cases and intermittent preventive treatment in pregnancy (IPTp), vector control is an important tool in the reduction of new cases. Alongside the use of long-lasting insecticidal nets (LLINs) and indoor residual spraying (IRS), targeting the vector larvae with biological larvicides, such as *Bacillus thuringiensis israelensis* (*Bti*) is gaining importance as a means of reducing the number of mosquito larvae before they emerge to their adult stage. This study presents data corroborating the entomological impact of such an intervention in a rural African environment.

**Methods:**

The study extended over 2 years and researched the impact of biological larviciding with *Bti* on malaria mosquitoes that were caught indoors and outdoors of houses using light traps. The achieved reductions in female *Anopheles* mosquitoes were calculated for two different larviciding choices using a regression model.

**Results:**

In villages that received selective treatment of the most productive breeding sites, the number of female *Anopheles* spp. dropped by 61% (95% CI 54–66%) compared to the pre-intervention period. In villages in which all breeding sites were treated, the number of female *Anopheles* spp. was reduced by 70% (95% CI 64–74%) compared to the pre-intervention period.

**Conclusion:**

It was shown that malaria vector abundance can be dramatically reduced through larviciding of breeding habitats and that, in many geographical settings, they are a viable addition to current malaria control measures.

## Background

Despite considerable achievements in its control, malaria is still one of the major burdens of disease in sub-Saharan Africa (SSA) and is responsible for an estimated 194 million cases and 346,000 death each year [1]. Alongside medical treatment, which is applied predominantly through the early diagnosis and treatment of malaria cases as well as intermittent preventive treatment in pregnancy (IPTp), the control of the transmitting vector is of paramount importance. The mostly applied vector control intervention by a wide margin is long-lasting insecticidal nets (LLINs), followed by indoor residual spraying (IRS).

Unfortunately, rapidly developing resistances, changing vector genetics and behaviour are decelerating or even reversing current gains in malaria control. Resistances are emerging to the widely used pyrethroid insecticides [2, 3], and there is increasing evidence for shifts in vector biting behaviour from night biting to early evening or early morning biting, and from indoor biting to outdoor biting [4–7]. In several places, the composition of the vector species was shown to change from previously predominantly indoor-biting *Anopheles gambiae* to predominantly *Anopheles arabiensis* and other vector species that prefer to bite and rest outdoors [8, 9]. However, the first-line malaria vector control tools almost exclusively target indoor-resting and indoor-biting mosquitoes and do not protect people when they spend time outdoors. There is urgent need for the additional implementation of interventions that are not compromised by the adaptations described above. Perhaps the oldest method of fighting mosquitoes is larval source management (LSM), the reduction of vector larvae via the elimination, transformation or treatment of larval breeding sites.

LSM has seen a respectable success in bringing down the number of vector mosquitoes as well as in reducing the number of malaria infections [10–12] and might regain some of its importance in the light of current developments. Despite its effectiveness and low operational cost in many settings, its deployment still lags far behind the use and general promotion of LLINs and IRS.

Promising candidates for scaled up applications are biological larvicides, such as *Bacillus thuringiensis israelensis* (*Bti*) and *Bacillus sphaericus* (*Bs*). Those larvicides are shown to have a strong lethal effect on larval populations, leading to reductions in adult vector densities, and have an impact that lasts between one and 2 weeks [13–19]. This paper, analysed the impact of biological larviciding with *Bti* on the abundance of malaria vector mosquitoes in a large-scale intervention trial in a rural health district in Burkina Faso. Evidence was generated, that environmental larviciding can lead to a major reduction of vector populations even in rural areas, where its deployment is considered more costly and difficult because of infrastructure and logistical limitations [20]. Additionally, the entomological impact of a cost-saving, risk map based larviciding application was evaluated. This study is unique in that it covers a complete health district in a mainly rural area and uses remote sensing based risk maps to detect larvae infested water bodies.

## Methods

### Study area

The intervention study was carried out in 127 rural villages and the semi-rural district capital of Nouna, located in the Kossi region of Northwestern Burkina Faso, covering an area of about 4770 km^2^ and 156,000 inhabitants. The majority of malaria transmission occurs during the rainy season from July to October, with a marked peak during August and September. The principal malaria vector in the study area is *Anopheles gambiae* sensu lato (s.l.), at more than 90%, followed by *Anopheles funestus* and *Anopheles nili* [5]. Within the study area insecticide-treated bed net coverage is very high, with 66% and 98% of young children sleeping under them during the dry and rainy season, respectively [21]. The utilization of IRS is virtually non-existent. Intermittent preventive treatment in pregnancy (IPTp), and early diagnosis and treatment of malaria cases with artemisinin-based combination therapy (ACT) are available [22], but, depending on the type of malaria manifestation, are in competition with traditional anti-malarial medicines to varying degrees [23]. Additionally, treatment success is often impaired by the use of sub-standard and fake medications [24].

### Study design

**Fig. 1:**
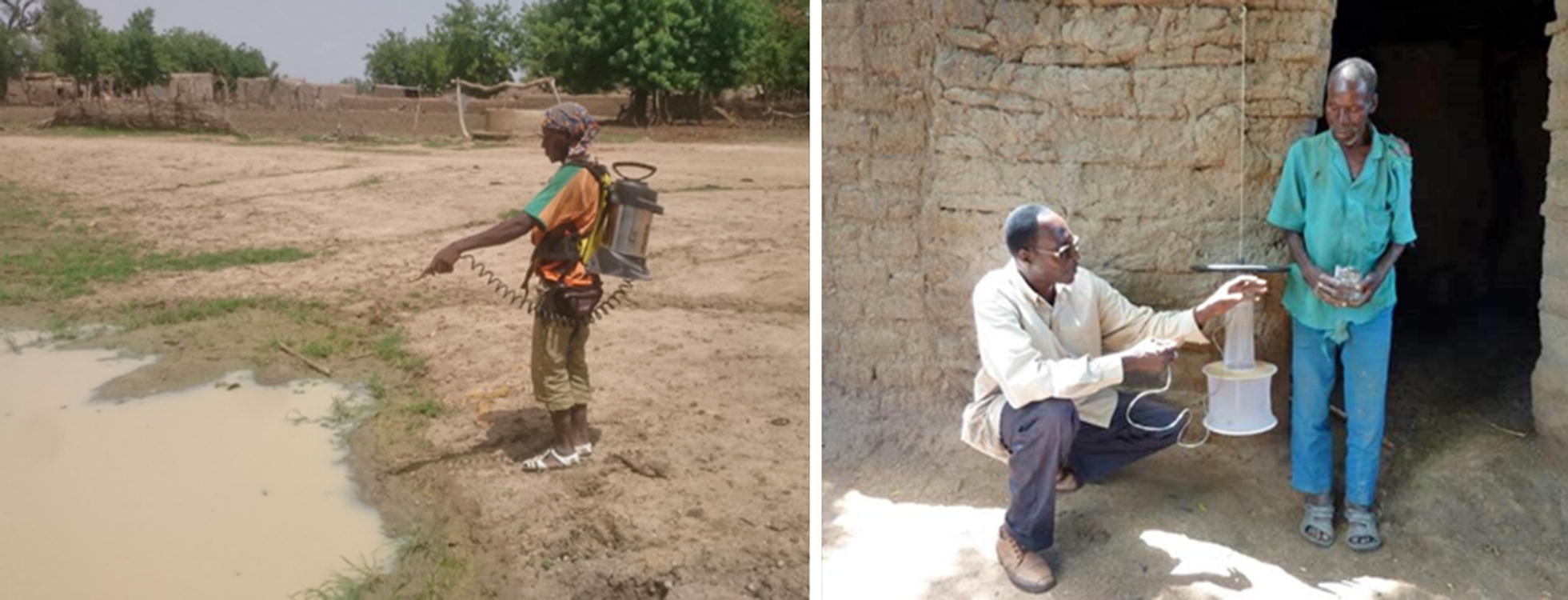
On the left: Spraying of mosquito breeding sites with Bti WG by trained members of the community using knapsack sprayers. On the right: CDC light trap being checked before installation within a rural village by an entomological technician

A total of 127 rural villages and the semi-urban town of Nouna were distributed into three study arms which received different larviciding choices: exhaustive treatment of all breeding sites (full treatment), guided treatment of only the breeding sites with the highest larval densities determined by remote sensing based risk maps, an untreated control group. To attribute for geographical differences such as surface water availability, vegetation, soil type, and precipitation, villages of each study arm were further distributed into three clusters, resulting in a total of nine clusters. A more detailed description about the study design can be found elsewhere [25]. The study covered a period of 2 years (2013–2014) with the first year representing the non-intervention baseline data collection within the whole area. During and after the rainy season of 2014 (July throughout October), larviciding with *Bti* VectoBac^®^ WG, AM65-52 strain (Valent BioSciences Corporation, IL, USA) was performed (Fig. 1, left side). Prior to the intervention, the optimum dosages for field application were identified [15]. Maps with all publicly accessible water bodies were generated during field visits for villages with exhaustive larviciding using GPS devices. For villages that received selective treatment, remote sensing derived risk maps of larval productivity were used [26]. Those risk maps are based on two types of information that were collected during field visits, densities of *Anopheles* larvae hat were seized through a standard dipping procedure, and water parameters that can be distinguished via remote sensing, such as water colour, turbidity, and different types of vegetation.

### Adult mosquito monitoring

Adult mosquitoes were collected using Center for Disease Control light traps (Model 512, John W. Hock Company, Gainesville, Florida) (Fig. 1, right side). Indoor and outdoor light trap captures were carried out in 27 villages in 2013 and in 36 villages in 2014, as well as in the seven town quarters of the district capital Nouna. Captures were carried out twice a month in a rotating system with two independent fieldwork teams, covering 4 villages per night, resulting in a total of at least 10 sample rounds per village per rainy season.

In each village and town quarter, three locations distanced approximately 100 to 150 m from each other were chosen for their central position in the village and in agreement with the household head. The traps were installed indoors near the sleeping places equipped with untreated bed nets and outdoors within the courtyard; they were positioned about 1 m above the ground. Mosquitoes were collected between 18:00 and 06:00 h to fully cover the biting period. Species determination was performed using microscopes, following the WRBU (Walter Reed Biosystematics Unit) identification keys [27].

### Statistical analysis

Statistical analysis was performed using Stata/IC 14.2 for Windows (StataCorp LLC, 4905 Lakeway Drive, College Station, TX 77845, USA). Female *Anopheles* counts collected per night per trap were used as the outcome variable. A negative binomial regression (Stata function “nbreg”), corresponding to a generalization of a Poisson distribution to account for the data over-dispersion, was performed. The random effect was integrated at village level.

## Results

**Fig. 2:**
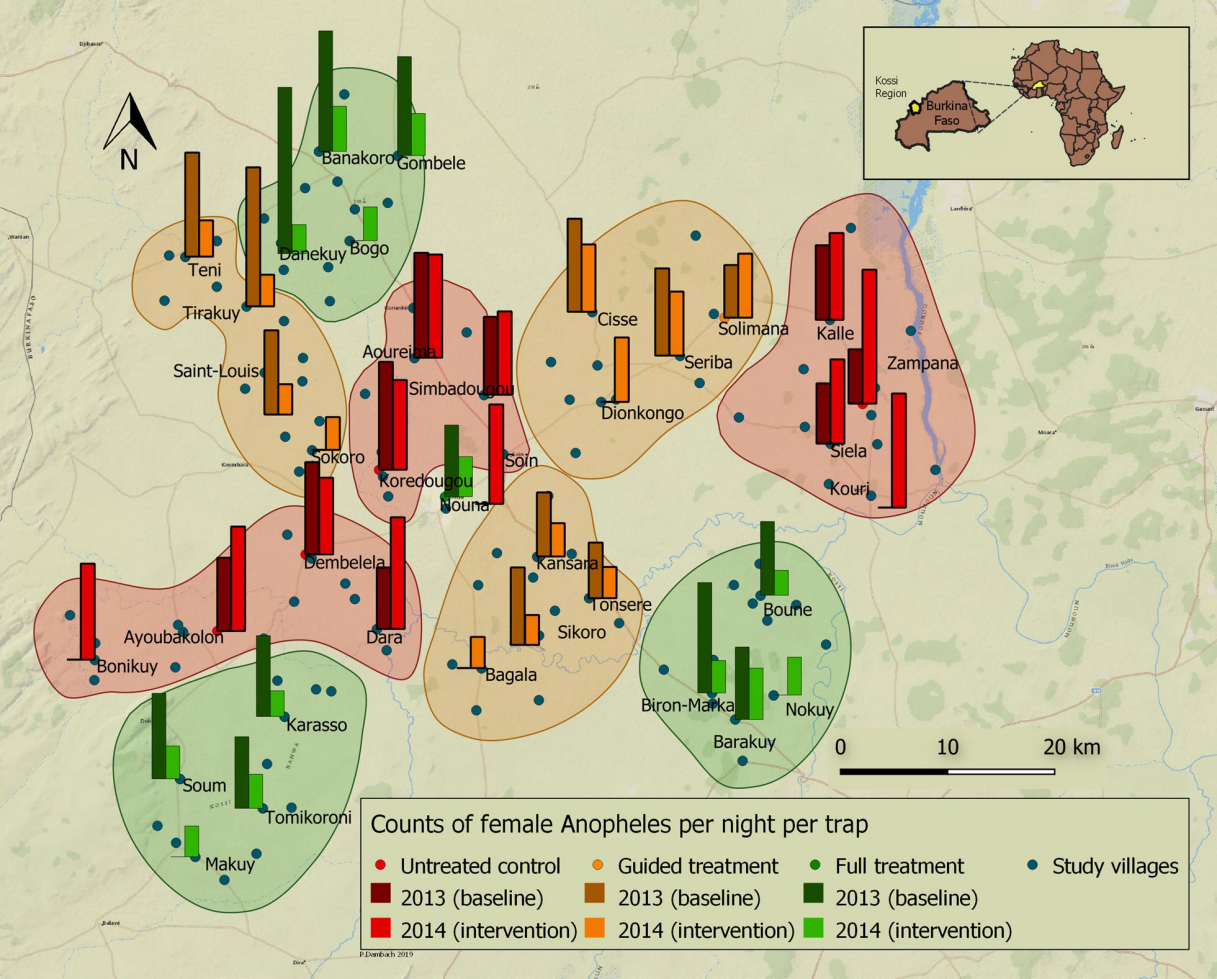
Study villages are shown with blue dots; bars indicate mosquito density in villages where mosquito captures took place. Bars show the average numbers of female Anopheles mosquitoes captured per trap per night indoors and outdoors using CDC light traps in September and October 2013 and 2014. Colours indicate treatment choice (Green = full treatment, orange = selective treatment, red = untreated control group). Closed lines encompass clusters of villages receiving the same treatment. In 2014, 9 additional villages were added to the mosquito collections

A total of 36,148 female mosquitoes were collected during the 2-year study period, of which 9022 (25%) were *Anopheles* spp. Among *Anopheles* spp. 89% were *An. gambiae* s.l. Figure 2 illustrates the spatial and temporal variation of mosquito abundance in the study region during baseline and intervention years. In the villages that served as the untreated control group, female *Anopheles* densities were on average 44% higher in 2014 compared to the baseline year 2013, indicating the generally higher natural mosquito abundance during the intervention year.

**Fig. 3:**
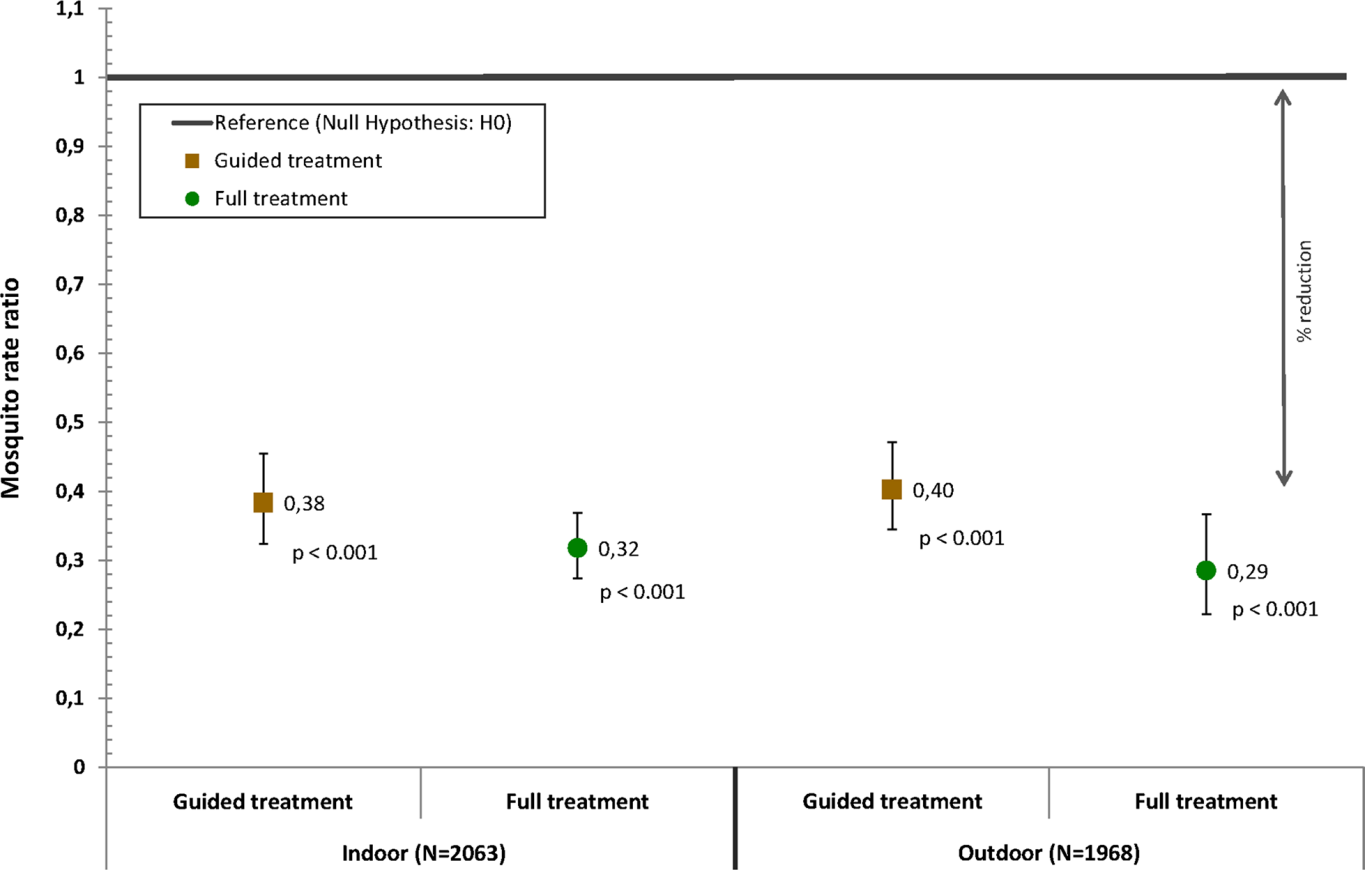
Point estimates of the regression model for the intervention year compared to the baseline year indicating the reduction in the count of indoor and outdoor female Anopheles mosquitoes per night per trap achieved through guided or full Bti treatment. The reference line represents the rate ratio value under the null hypothesis: i.e. the count of female Anopheles mosquitoes in the control areas receiving no Bti treatment are not different from the counts in areas receiving guided or full Bti treatment

Larviciding with *Bti* reduced the mean densities of female *Anopheles* mosquitoes during the intervention year in both treatment arms (Fig. 3). Within villages that received selective treatment of only the most productive breeding sites using remote sensing based risk maps, the number of female *Anopheles* spp. caught during light trap captures dropped by 62% (95% CI 54–68%) indoors and 60% (95% CI 53–66%) outdoors compared to the pre-intervention period. In villages in which all breeding sites were treated, the number of female *Anopheles* spp. was reduced by 68% (95% CI 63–73%) indoors and 71% (95% CI 63–78%) outdoors compared to the pre-intervention period (Table 1).

**Table 1:** Negative binomial regression model comparing mosquitoes counts in villages treated with three different Bti-interventions during non-intervention (2013 baseline) and intervention (2014) periods for female Anopheles captured indoors and outdoors, analysed together and separately. The random effect was integrated at village level

*Bti* interventions	N	Incidence-rate ratio (95% CI)	*p*	% reduction (95% CI)
All (N = 4031)				
Untreated control	2973	–		
Guided treatment	409	0.393 (0.340–0.455)	< 0.001	60.7% (54.5%–66.0%)
Full treatment	649	0.304 (0.258–0.359)	< 0.001	69.6% (64.1%–74.2%)
Indoors (N = 2063)				
Untreated control	1522	–		
Guided treatment	208	0.384 (0.324–0.455)	< 0.001	61.6% (54.5%–67.6%)
Full treatment	333	0.318 (0.274–0.369)	< 0.001	68.2% (63.1%–72.6%)
Outdoors (N = 1968)				
Untreated control	1451	–		
Guided treatment	201	0.403 (0.345–0.471)	< 0.001	59.7% (52.9%–65.5%)
Full treatment	316	0.285 (0.222–0.367)	< 0.001	71.5% (63.3%–77.8%)

**Fig. 4:**
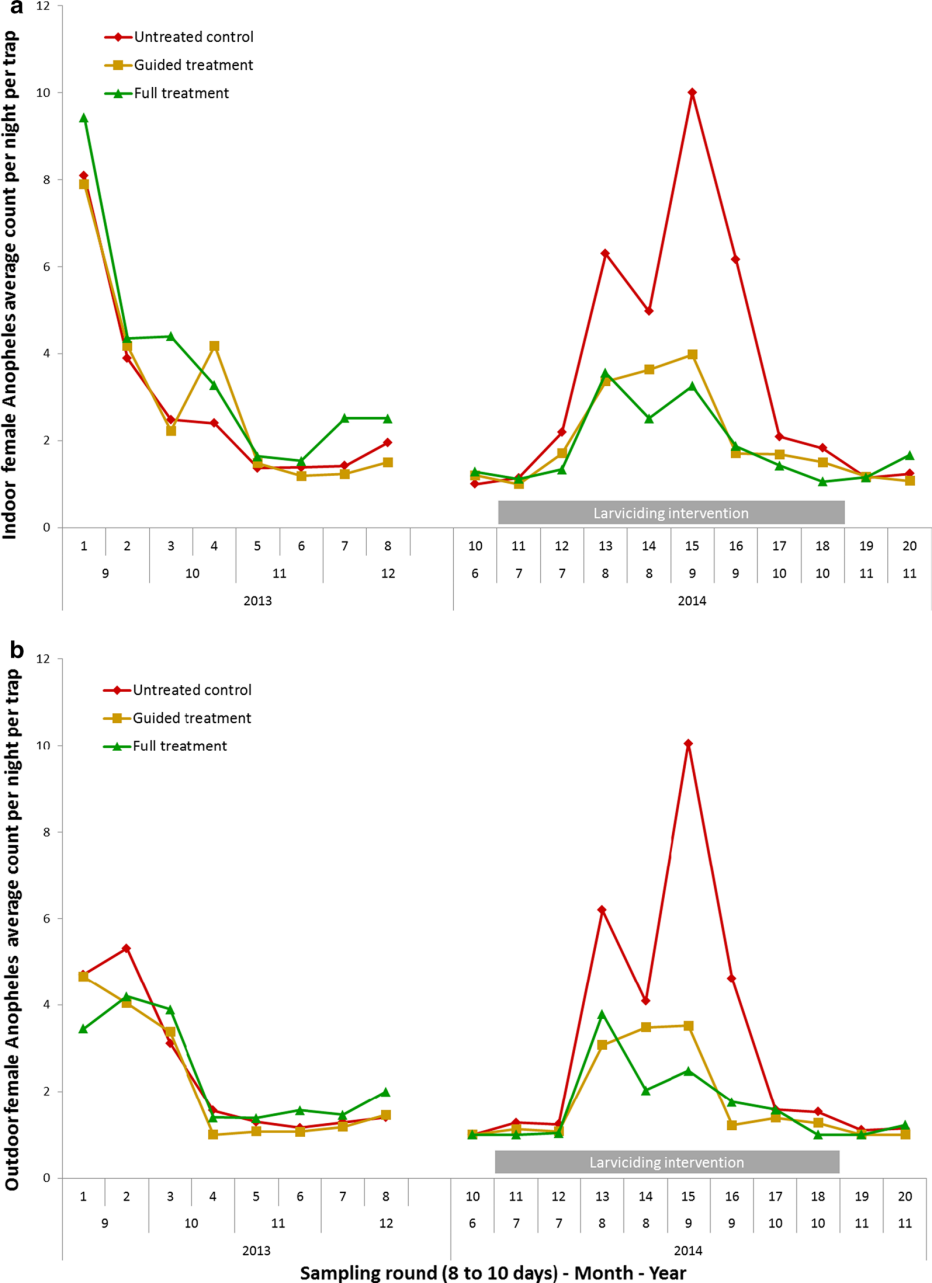
Average numbers of female Anopheles mosquitoes per trap per night caught **a** Indoors and **b** outdoors during the successive sampling rounds of the study period. The different Bti treatments are colour coded. The duration of performed larviciding is indicated with gray bars

Figure 4 shows the timeline of female *Anopheles* mosquito counts during the successive sampling rounds of the study and seasonal variation of mosquito abundance. In 2014, the month with the highest achieved mosquito reduction was September, which at the same time featured the highest mosquito abundance. Mosquito densities were reduced by 75% (95% CI 71–79%) indoors and 79% (95% CI 72–85%) outdoors in the full treatment study arm and by 73% (65–80%) indoors and 72% (63–79%) outdoors in the guided treatment arm compared to the pre-intervention period. To capture the epidemiological importance of reductions it is crucial to consider the absolute numbers. Mosquito reductions of 68% in the full treatment arm in September 2014 represent a drop from 7.2 to 2.3 female *Anopheles* per night per trap, while the 29% drop in October 2014 indicates a reduction from an already relatively low level of 1.8 down to 1.3 mosquitoes per night per trap.

## Discussion

This study showed that malaria vector abundance in rural Burkina Faso was largely reduced through *Bti* based larval source management. Despite the rural nature of the survey region, which is sometimes seen as an argument against the cost-effectiveness of larviciding, high mosquito reductions were achieved through a treatment of all detected and publicly accessible mosquito breeding sides at per capita costs of US$ 1.05 per year. The guided treatment approach using satellite derived risk maps realized the intervention at lower costs of US$ 0.77 [28], but did not lead to the same reductions in adult *Anopheles* mosquitoes. Those values are comparable with findings from other studies, which ranged between US$ 0.90 and 2.50 [11, 29, 30] and are slightly below the annual costs for indoor residual spraying in the region [31] and those for LLINs and conventional insecticide-treated nets [32].

There is heterogeneity in the reductions achieved between studies. The limited number of studies available, the variety in impact evaluation indicators, different sampling techniques and environmental settings make it difficult to compare the observed full treatment reductions of 68% indoors and 72% outdoors with those from other studies. Furthermore, the study setup included a guided treatment choice which is novel and cannot be compared with other studies. While some studies reported the change in entomological inoculation rates (EIR—the number of infective bites per person per time, usually per year), others used general vector abundance measured through human landing catches (HLC), resting stations, pyrethrum spray catches or light traps. Studies that applied EIRs reported reductions of 21.3% [33], 31.5% [34], and 84.6% [35]. Two studies reported both, EIRs and data from resting stations. Fillinger et al. [10] found reductions of 73.0% (EIR) and 85.9% (resting stations). Coulibaly [35] described in Tusting et al. [12], reported reductions of 84.6% (EIR) and 37.8% (resting stations). A study in the Gambia did not achieve reasonable reduction, mainly due to the vastly inundated areas of the river Gambia [18]. The mosquito reductions achieved with guided treatment, which treats only 50% of breeding sites that harbored the highest larval infestations determined through risk maps, were found to be on average 9% lower compared to a full treatment of all breeding sites in and around villages. This lower impact on vector abundance relates to cost savings of about 27% [28]. Although a guided treatment approach seems to be advisable in terms of effective resource allocation in this particular setting, these findings might not have universal validity for other regions. Generally, and from an epidemiological point of view, the safest and easiest way to reduce vector mosquito abundance is a full treatment scenario.

It is important to keep in mind that the number and extent of larval sources and adult mosquito densities within the survey region are highly heterogeneous. While a large proportion of villages showed only a very limited number of breeding grounds, such as anthropogenic waterholes for cattle, other areas are influenced by riverine systems with vast flooded areas. Mosquito densities equally varied by village. This leads to the conclusion that the assessment of an area´s appropriateness for larviciding can be seen as a form of mixed calculation, being more expensive in some villages than in others. This is equally true for the achieved mosquito reductions throughout the year, with the high transmission season showing higher possible reductions. The determining basis for cost–benefit calculations should hence be seen as an average over an administrative area rather than estimations from single villages.

### Strengths and limitations

Strengths of this study include its large spatial and temporal extent of larviciding activities in 127 rural villages and a semi-rural town over the period of 3 years. Equally the amount and collection frequency of entomological data is extensive compared to many other studies. There are also limitations to this study. The collection of mosquito abundance data in 2013 started later than initially planned and is available from September on only. This leads to a relatively short overlap period of 3 months with the mosquito sampling of the following intervention year. The randomization of treatment arms was done at the level of village clusters. Although this does not correspond to the standard approach for a randomized control trial (RCT) of a medical study, it was the best possible approach in a geographical and environmental context. Because mosquitoes not only bite in the immediate vicinity of their breeding grounds but travel some distance during their search for blood meal, larviciding is ideally applied over a larger area to avoid infiltration of mosquitoes from untreated areas. For this reason, villages in which the same larviciding approach was applied were intentionally geographically clustered.

### Deductions for malaria control policies

The results of this study bear implications for malaria vector control in rural sub-Saharan Africa. The findings presented here are congruent with those from some other studies in SSA and contrast the current WHO recommendation that “larviciding should be considered for malaria control (with or without other interventions) only in areas where the larval habitats are few, fixed and findable” [12, 33, 34, 36, 37]. While for good larviciding success it is important that larval sources are “findable”, it seems that it is not an absolute prerequisite that they are “few” or “fixed”. On the other hand, limitations for the cost-effective use of biological larviciding arise from geographical and climatic conditions, such as the length of the rainy season, including its bimodal pattern in regions close to the equator. A prolonged rainy season requires an extension of the treatment period, which results in increased intervention costs [28]. Another factor that can impede larviciding success or even render it impossible is the existence of large inundated areas as it was shown in the floodplains of The Gambia [18]. For the assessment of intervention costs, the heterogeneity of a target region needs to be taken into consideration. Within the study area in Burkina Faso, several larger inundated areas such as wet rice fields were requiring high amounts of larvicide and workforce, while in other villages with very few water bodies only little costs accrued, leading to an equalization, when applying a holistic view, averaging over all study villages. The WHO aims to define universally valid recommendations and has to orchestrate a panoply of different vector control measures and assure a cost-effective application of funds. This global strategy has advantages because it canalizes available means and avoids the deployment of less promising vector control measures. The downside of a selective policy with a rather limited recommended range of application is that it impedes the implementation of new approaches even when the needed funds would be raised by other donors or on a community basis. In light of the results demonstrating the effectiveness of larviciding in rural areas, the WHO’s current policy of recommending biological larviciding only for urban areas should be reevaluated. Rural areas with a limited number of larval sources are numerous, particularly in the Sahel, and might indeed be appropriate for environmental larviciding even if population density is low. Malaria control might benefit from a more flexible approach of assessing the technical feasibility and cost-effectiveness of biological larviciding in a region and equally a more flexible way of recommending or approving biological larviciding in an extended set of selected settings. This might facilitate the generation and use of resources from national anti-malaria campaigns or the acquisition of funding from external donor organizations.

## Conclusions

Adding LSM to existing malaria control programmes is an effective approach to reduce malaria vector abundance and hence human exposure. Despite the vast terrain and low population densities in many rural areas, *Bti* based larviciding is a powerful tool that can be applied with moderate effort. The general advantages of larviciding over adult vector control, namely exposure reduction around the clock and facile accessibility of vector larvae, are valid for rural environments as they are for urban ones, although it is evident that with current *Bti* formulations and application techniques not all areas are eligible for treatment. In the light of evolving parasite strains and increasing resistance of mosquitoes towards anti-malarial drugs [38, 39] and pyrethroid insecticides [40–42], LSM should be reevaluated as a standard tool for additional vector control in suitable areas. Equally for areas where classical malaria control methods have achieved considerable success, LSM can be a tool to maintain achieved reductions. LSM should not be considered a stand-alone intervention but when added onto the currently implemented control measures it could largely increase the success of malaria vector control.

## Data Availability

The datasets supporting the conclusions of this article are available at the Health Research Center in Nouna, Burkina Faso and will be made easily available on request, when required.
